# Biomedical applications of solid-binding peptides and proteins

**DOI:** 10.1016/j.mtbio.2023.100580

**Published:** 2023-02-15

**Authors:** Nicolò Alvisi, Renko de Vries

**Affiliations:** Physical Chemistry and Soft Matter, Wageningen University & Research, Stippeneng 4, 6708WE, Wageningen, the Netherlands

**Keywords:** Solid-binding peptides, Solid-binding proteins, Biomaterials, Bioconjugation, Biomedicine, Surface coatings, Implants, Biosensing, Protein design, Adsorption-based coatings

## Abstract

Over the past decades, solid-binding peptides (SBPs) have found multiple applications in materials science. In non-covalent surface modification strategies, solid-binding peptides are a simple and versatile tool for the immobilization of biomolecules on a vast variety of solid surfaces. Especially in physiological environments, SBPs can increase the biocompatibility of hybrid materials and offer tunable properties for the display of biomolecules with minimal impact on their functionality. All these features make SBPs attractive for the manufacturing of bioinspired materials in diagnostic and therapeutic applications. In particular, biomedical applications such as drug delivery, biosensing, and regenerative therapies have benefited from the introduction of SBPs. Here, we review recent literature on the use of solid-binding peptides and solid-binding proteins in biomedical applications. We focus on applications where modulating the interactions between solid materials and biomolecules is crucial. In this review, we describe solid-binding peptides and proteins, providing background on sequence design and binding mechanism. We then discuss their application on materials relevant for biomedicine (calcium phosphates, silicates, ice crystals, metals, plastics, and graphene). Although the limited characterization of SBPs still represents a challenge for their design and widespread application, our review shows that SBP-mediated bioconjugation can be easily introduced into complex designs and on nanomaterials with very different surface chemistries.

## Graphical abstract

**Figure:**
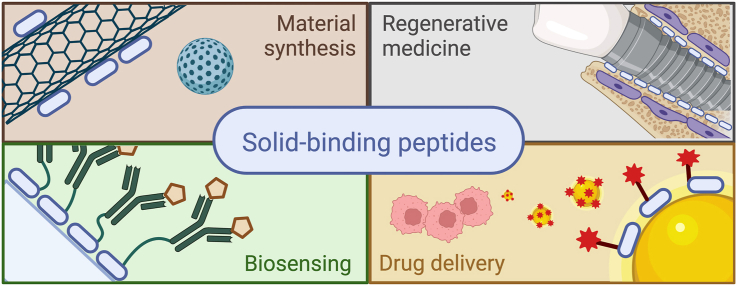

## 1 Introduction

For over 3.7 billion years, living organisms have evolved strategies to survive in challenging environments [1]. One of the earliest strategies that organisms adopted for survival was the manipulation of inorganic solids in their environment. In particular, the incorporation of inorganic solid materials in organized extracellular architectures allowed for the creation of solid structures for defense, support, and spatial organization [2]. Early in the evolution of multicellular organisms, silica and calcium carbonate were already harvested and organized in spicules by sponges or mother-of-pearl by mollusks, in a process known as biomineralization [3,4]. Next to biomineralization, living organisms have also evolved strategies for interacting with solid surfaces in their surroundings, for example as a means of colonizing specific environments such as rocks or ice [5,6].

Organisms have at their disposal a plethora of mechanisms for the recognition and binding of inorganic surfaces. Specifically, proteins and peptides prominently feature among the biomolecules that mediate these interactions. Many proteins involved in solid-binding and biomineralization have been described in recent years, providing insights in the interfacial mechanisms of solid recognition [7]. Notable examples are lustrin A, responsible for binding the matrix of shell and pearl nacre [8], silaffin-2, produced by diatoms in the matrix of biosilica [9], and the proteins involved in the self-assembly of magnetite crystals inside magnetosensitive bacteria [10,11].

The synthesis of novel biomaterials has taken much inspiration from natural proteins that interact with solid surfaces. The integration of inorganic solids in a biological environment is essential in the development of effective medical devices such as implants, catheters, and prosthetic joints [12]. Furthermore, many bioanalytical devices rely on receptors attached to solid surfaces. Aside from silicates and hydroxyapatite, new materials such as titanium, gold, silver, and plastics have been utilized for the development of biomedical devices. While these materials can be used without further modifications, undesired interactions with biomolecules, cells, and tissues can hinder their successful and long-term use [13]. It is therefore clear that novel biomaterials must precisely control the interactions of biomolecules with solid surfaces.

The most common functionalization approaches are based on the physical and chemical modification of biomaterial surfaces, like grit-blasting and acid etching of titanium implants [14]. These methods are widely applied given their scalability and robustness, although they are still lacking with regards to biocompatibility. Alternatively, biofunctionalization is achieved via the immobilization of synthetic coatings such as self-assembled monolayers (SAMs) or the multilayer deposition of various polymeric materials [15,16]. These strategies are robust and versatile since they rely on the covalent immobilization of selected biomolecules [17]. Nevertheless, there is increasing interest in employing nature's toolkit by using solid-binding peptides and proteins. As previously mentioned, the recognition and binding of solid surfaces via non-covalent interactions is an effective strategy adopted by many organisms. The use of solid-binding peptides (SBPs) can improve the biocompatibility of surface functionalization strategies, allowing for the display of a wide range of biologically active macromolecules while retaining their function, with high surface specificity and ease of use.

**Fig. 1:**
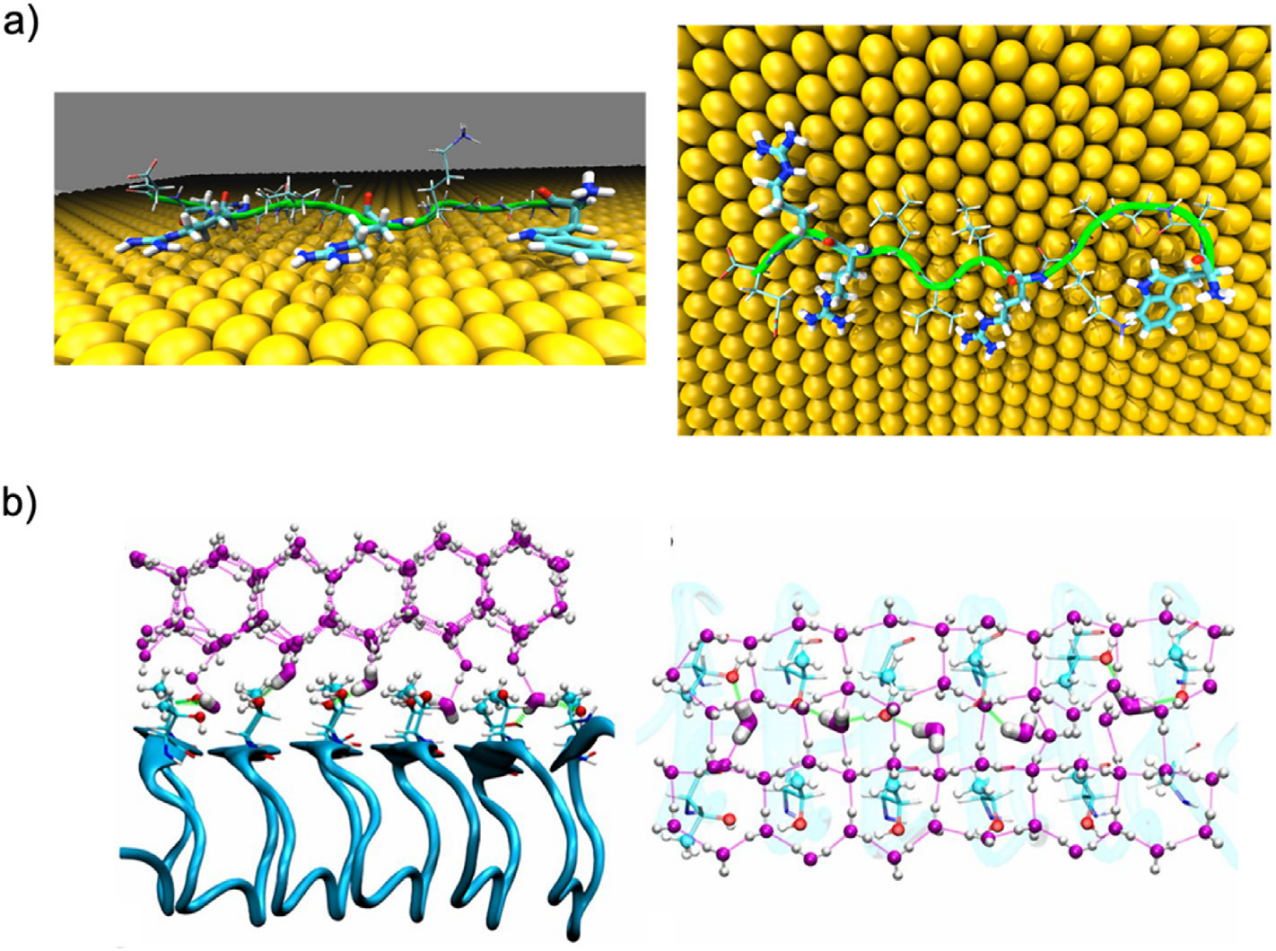
Molecular dynamics simulations of solid-binding peptide and solid-binding protein. a) Typical disordered structure for the surface adsorbed conformations of the gold-binding peptide AuBP1. Side view on the left, top view on the right. The amino acid residues interacting with the gold surface are highlighted with thicker bonds. Water molecules are not shown for clarity. Reprinted with permission from Ref. [23]. Copyright 2013 American Chemical Society. b) Simulation results for the structured antifreeze protein *MpdAFP* of the beetle *Micromeria punctipennis dzungarica*. Side view (left) and top view (right) of the hexagonal ice-like water molecules atop the ice-binding site of the protein. The ice-binding site consists of an ordered array of β-sheets (blue arrows). Reprinted with permission from Ref. [24]. Copyright 2016 National Academy of Sciences.

Solid binding peptides (SBPs) are short amino acid sequences that can specifically recognize and bind a vast range of solid surfaces. In general, SBPs bind to solid surfaces via multiple non-covalent interactions. Various factors can define the binding strength of SBPs, such as surface topography and solution conditions. However, a key aspect of binding solid surfaces resides in the amino acid composition and the structural variety of SBPs. The term “solid-binding peptide” is usually reserved for short and unstructured peptides, typically with disordered conformations both in solution and when adsorbed on flat surfaces (Fig. 1a). For the purpose of this review, we distinguish these SBPs from longer solid-binding domains or solid-binding proteins that may adopt ordered three-dimensional structures (Fig. 1b) [18,19]. It is nonetheless worth mentioning that short SBPs lacking a secondary structure can still exhibit preferred binding conformations on flat surfaces. Ice-binding proteins (IBPs) are a notable example of the natural variability of interfacial recognition structures. IBPs have independently evolved in several biological kingdoms, resulting in a large size range (3–180 ​kDa) and different physiological roles [20]. Most importantly, they present a remarkable variety of structural organization, from short glycosylated peptides to three-dimensional rigid structures like parallel α-helices, four helix bundles, and β-solenoids [21,22]. These examples highlight how the lack of a precise secondary or tertiary structure is not necessary for binding solid surfaces, both in crystalline and amorphous solids.

The advantages introduced by SBPs in bioconjugation methods are especially attractive for biomedical research. Firstly, biologically relevant surfaces can be functionalized more easily compared to conventional bioconjugation techniques. For example, direct coupling reactions (sulfo-NHS, EDC) or SAMs require multiple steps with harsh reagents, while standard molecular biology skills and equipment are sufficient for the use of SBPs. Secondly, the production of recombinant proteins is already a highly scalable industrial technique. Also, SBPs offer a great control over the directionality and orientation during the immobilization of the biomolecules. Considering that conformation and orientation are crucial for maintaining biological activity, SBPs are a safe alternative. Lastly, the use of nature-inspired peptides guarantees improved biocompatibility in biological surroundings.

Over the years, the increasing amount of evidence surrounding the application of SBPs has brought attention to the topic. Given the broad spectrum of possible applications within materials science, SBPs have created many opportunities for novel bioinspired materials. In this review, we provide an overview of recent literature on the use of solid-binding peptides, solid-binding domains, and solid-binding proteins in biomedical applications. We focus on applications where modulating the interactions between solid materials and biomolecules is crucial. While we include few examples relevant for biomineralization, we mainly concentrate on applications featuring pre-existing, untreated solid surfaces. Furthermore, we find that most applied work makes use of short and unstructured SBPs rather than of larger folded solid-binding proteins, presumably due to the ease of obtaining and including short peptides in larger molecular architectures. Reflecting this difference, we mainly focus on SBPs, while briefly addressing globular solid-binding domains and proteins.

In recent years, other comprehensive reviews on SBPs have been published. On the one hand, most of them have a broad focus and only include discussions of biomedical applications, as one of the many application areas of SBPs [25,26]. Pushpavanam et al., for example, explored the application of SBPs as molecular linkers and material synthesizers [27]. On the other hand, other authors reviewed biofunctionalization techniques for specific biomedically-relevant surfaces, without a clear focus on SBPs [[28], [29], [30], [31]]. Only Care et al. provided a brief overview on the use of SBPs as tools for medically-relevant solid interfaces and biomaterials [32]. With our review, we hope to provide a more extensive and updated analysis.

In the first part of this review, we provide background on SBP sequences and on the molecular mechanisms of their binding to solid surfaces. Next, we review the biomedical applications of SBPs to solid surfaces relevant for biocompatibility and biosensing, such as calcium phosphate minerals, silica, ice, metals, metal oxides, plastics, and graphene. In each case, we focus on studies that describe how SBPs are successfully integrated in larger designs with biomedical applications. In the outlook, we provide suggestions for future work and opportunities in this area.

## 2 Background

### 2.1 Design of solid-binding peptides and proteins

Natural adhesion peptides and proteins are products of the evolutionary process. Consequently, these peptides may not have the required properties for biomedical applications. Moreover, no specific binders have evolved in nature for some crucially important synthetic biomaterials, such as titanium and plastics. Hence, design of new adhesion peptides and adhesion proteins is an important issue in materials science [33].

**Fig. 2:**
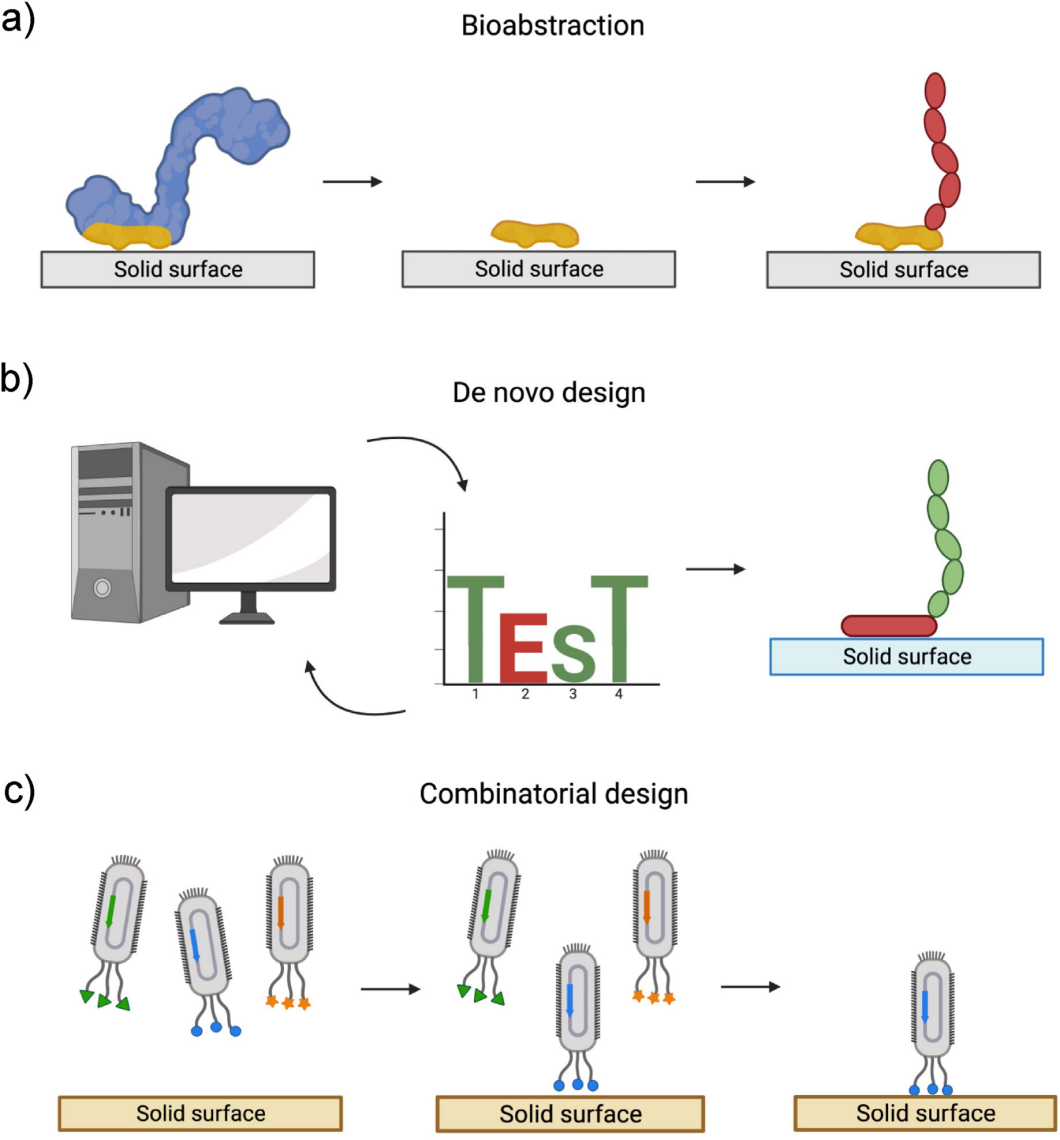
Examples of different peptide identification and design strategies. a) In bioabstraction, functional domains are isolated from naturally occurring proteins. b) Rational and computational methods can be applied, also to existing sequences, for the design of de novo peptides with the desired binding characteristic. c) Phage display is one of the many combinatorial methods used for the discovery of functional peptides. A library of bacteriophages, which display a certain peptide sequence, is used for “biopanning” and enrichment on a surface of interest. Strongly bound bacteriophages contain the genetic information for the synthesis of high affinity peptide sequences.

Three main strategies have been used to identify and design functional peptide sequences for material binding: bioabstraction, de novo computational design and combinatorial design. While bioabstraction and combinatorial design have typically led to sequences for SBPs, de novo computational design has so far mainly been successful for the design of solid-binding proteins. The three strategies are illustrated in Fig. 2.

First, bioabstraction involves the isolation of solid-binding regions, usually from natural proteins (Fig. 2a). The increasing availability of three-dimensional protein structures has allowed the discovery of minimal functional domains that were successfully adapted for surface functionalization. The unexpected discovery of micro-organisms capable of binding and degrading synthetic polymers has expanded the pool of possible surfaces [34,35]. Nonetheless, this technique has proven to work only in some specific cases [[36], [37], [38]]. In fact, the evolutionary pressure on organisms to select efficient binders for synthetic surfaces has obviously been very limited. For this reason, bioabstraction is mostly limited to surfaces with which organisms naturally interact.

Next, de novo computational design entails the generation of synthetic proteins with amino acid sequences that are not found in nature, based on biophysical and biochemical principles [39] (Fig. 2b). Considerable advancements can be attributed to improved computational methods and computing power, as well as synthetic manufacturing of new DNA sequences. De novo computational design of proteins is a well-established method and first attempts have been made in the design of folded proteins that bind to specific crystalline surfaces [[40], [41], [42], [43], [44], [45]]. First principles computational design is currently less successful for disordered SBPs, although the first steps have been made [46]. However, it remains difficult to rationally design binders for materials with no clearly defined structure due to, for example, variations in local curvature, crystal plane morphology, topology, and crystal defects [47]. Despite recent successes, these methods have so far been mainly employed towards small molecule binders and not solid materials [48].

Finally, the most used and most successful method is combinatorial design. Combinatorial design involves the synthesis of peptide libraries and the subsequent screening and selection for binding to the surface of a specific solid material. This technique was introduced by George Smith as phage display, for which a library of bacteriophage viral particles is used to screen the peptides [49]. In phage display, random peptide sequences are fused on specific viral coat proteins, so that each viral particle displays several copies of the same peptide (Fig. 2c). With multiple rounds of “biopanning” and multiplication, the phage library is enriched in peptides with the desired binding properties: after incubating phages with the material of interest, the weakly bound phages are washed away while the strongly bound phages are eluted separately. The eluted phages can be replicated and sequenced to identify the peptide. With this method, multiple solid-binding peptides can be identified, with high affinity for a wide variety of surfaces of interest.

Other methods of surface display have been developed, which use bacteria and yeast for cell surface display [50,51]. Phage display, however, shows fewer technical limitations and has been applied for the development of new drugs [52] and vaccines [53]. Furthermore, phage display is not limited to naturally occurring surfaces: strong binders have been identified for, among others, cellulose surfaces [54], metal oxides [55,56], titanium [57], platinum [58], hydroxyapatite [59,60], noble and rare earth metals [61,62], and polymeric materials [[63], [64], [65], [66]]. In a few cases, phage display has also allowed for the identification of solid-binding peptides capable of discriminating between different surfaces and even crystal planes of the same material [58,59,[67], [68], [69], [70]]. Unlike the previous design strategies, phage display can probe a very large chemical space and address defects and curvature of the solid surface. The resulting interactions generally yield more robust binding at different environmental conditions. Phage display, however, shows a clear bias for electrostatic bonds, generally resulting in low selectivity for the tested surface. For this reason, the sequences found via phage display often have little resemblance with known peptide motifs and are difficult to translate to empirical design strategies. Furthermore, since strongly bound phages display multiple copies of the same peptide sequence, surface functionalization strategies relying on a single peptide can be unsatisfactory. For an extensive overview of the use of phage display in materials science, we refer to Seker et al. [71].

In combinatorial design, peptide libraries can also be created synthetically and subsequently tested for the identification of SBPs. For example, in split-and-mix and SPOT libraries, peptides are synthesized on a solid support and then incubated with particles of the material of interest to identify sequences with strong binding [33,72]. Both split-and-mix and SPOT libraries allow to test fewer peptide combinations compared to phage display, but they provide more reliable results. A last promising alternative is directed protein evolution. This combinatorial technique is aimed at tailoring protein properties to specific application conditions. Starting from a known peptide with suboptimal properties, directed evolution iterates cycles of random mutagenesis and screening for improved protein variants with the desired characteristics [73]. Although directed evolution has been explored for a wide range of applications, its use in materials science is still in its infancy [[74], [75], [76], [77]].

### 2.2 Techniques for analysis of binding

The screening of combinatorial libraries is an effective way to find sequences for SBPs with high affinity for materials of interest, but it provides little information regarding binding mechanisms and strengths. Binding of peptides and proteins to solid surfaces is a complex physical-chemical process depending on various conditions, such as the chemistry of the surface, the composition of the solvent, the concentration of solutes, and temperature. Furthermore, only few methods are available for experimentally elucidating the atomic structures of peptides and proteins at solid interfaces, especially for disordered peptides. Unsurprisingly, the analysis of protein-surface interactions is a broad field of study, and we suggest the review by Bansal et al. for a more detailed discussion [78].

A straightforward characterization of SBPs can be performed by measuring the kinetics of adsorption and desorption of the peptides under a representative set of conditions. Many instruments are available for this type of analysis. For example, quartz crystal microbalance with dissipation monitoring (QCM-D) is becoming increasingly popular, since many solid surfaces are commercially available for testing and sensors can be easily modified. QCM-D uses a piezoelectric sensor that monitors mass changes on its surface. These data are often analyzed in terms of simple equilibrium models where peptides independently adsorb and desorb from the surface. However, this assumption is not always applicable, since some SBPs have been shown to assemble cooperatively on solid surfaces [79,80]. The recording of dissipation energy can provide additional information on the density and packing of the adsorber layers, especially in relation to changes in the surrounding solution (pH, ionic strength) [81].

Atomic force microscopy (AFM) in air is an imaging technique with nm resolution used for the visualization of SBP-coated surfaces. AFM imaging is an effective tool to study conformation, surface coverage, aggregation, and selectivity. This technique can therefore help establish if SBPs adsorb independently forming uniform coatings, or if they cooperatively assemble on the surface creating fibers and aggregates [79,80,82]. The formation of peptide layers can also be followed in real-time and in solution via AFM imaging, although the setup optimization is more laborious [40,83,84]. AFM can also be used for single-molecule force spectroscopy (SMFS) in order to measure the adhesion forces of a single SBP molecule to a solid substrate with pN accuracy [85]. A combination of these techniques can provide a reasonably thorough characterization of SBP binding. For example, a recent publication by our group showed how contrasting results from SMFS and QCM-D can be rationalized using AFM imaging [79]. In addition, our results highlighted the risk of characterizing SBPs in terms of a single equilibrium binding constant, since independent adsorption and full equilibrium cannot be assumed for all SBPs.

Finally, nuclear magnetic resonance (NMR) spectroscopy and solid-state NMR allow to study the structure and surface arrangement of SBPs both in solution and adsorbed to a surface, highlighting which side chains interact with the surface. Solid-state NMR is one of the few techniques providing structural information at the atomic level on the conformation of peptides adsorbed on solid surfaces. Unfortunately, these analyses are difficult and rarely applied for SBP characterization [78].

### 2.3 Molecular features determining binding of SBPs to solid surfaces

**Fig. 3:**
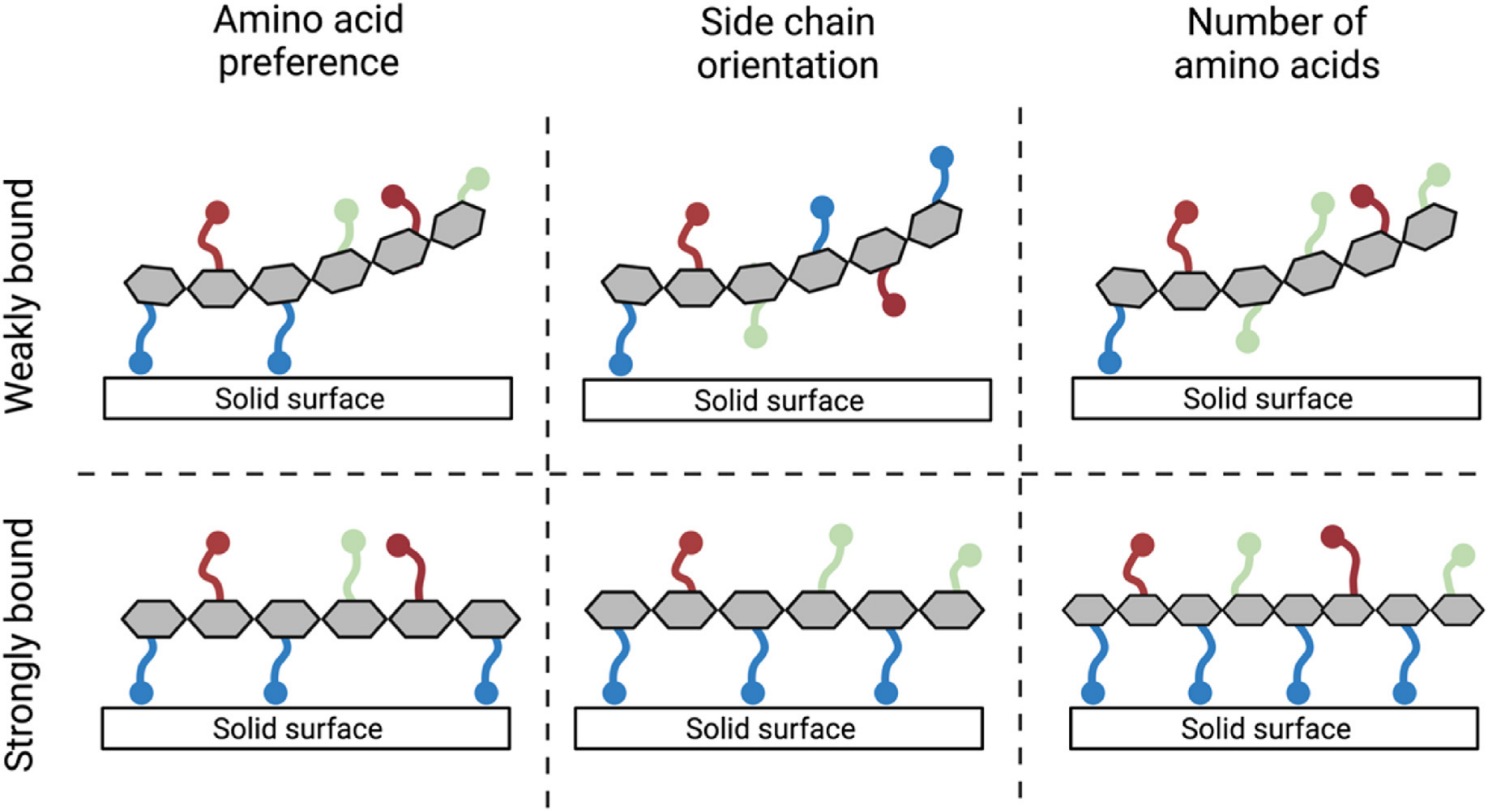
Schematic representation of selected molecular features that determine surface binding. Amino acids interact with solid surfaces via their side chain (in colour). Enrichment in amino acids with desirable chemical properties (charge, polarity, hydrophobicity) can highly influence the binding strength of a peptide sequence. The relative position and orientation of the side chains is also responsible for stronger binding. Finally, increasing the number of binding moieties can result in improved binding affinity.

Various mechanisms are involved in the recognition, binding, and affinity of SBPs to solid surfaces. Among others, we wish to point out three key molecular features that determine the binding of solid-binding peptides and proteins to solid surfaces: the amino acid composition of the binding region, the orientation of the amino acids in contact with the surface, and the number of amino acids in contact with the surface. A schematic illustration of these selected molecular features is presented in Fig. 3.

First, the amino acid composition can influence the binding strength, since the amino acid side chains contain chemical groups with affinity for certain solid surfaces (Fig. 3). For example, metal-binding SBPs are enriched in hydrophobic and polar residues, while positively charged amino acids significantly contribute to silica binding, and aromatic residues preferentially bind carbon-based materials [23,79,86,87]. Furthermore, threonine is crucial in most ice-binding proteins, and acidic amino acids strongly interact with calcium phosphate minerals, while peptides binding to polymeric materials are enriched in hydrophobic residues [76,88,89]. Single-residue mutants can provide insightful information on which amino acid residues contribute to the binding affinity of solid-binding peptides and proteins [90,91]. Each amino acid is systematically substituted with a “non-functional” amino acid like alanine, and the resulting peptides are tested with respect to their surface binding capabilities. Furthermore, these mutants show that the position of an amino acid within an SBP can highly affect the overall binding ability [92,93].

Second, the possible orientations of each residue with respect to the surface is a fundamental factor determining the binding strength of solid-binding peptides, domains, and proteins (Fig. 3) [94]. Short SBPs usually lack a stable three-dimensional structure, and their inherent flexibility allows them to adopt multiple conformations upon adsorption. In some cases, molecular simulations have shown that the orientation of anchor residues plays a key role in determining the overall binding strength [23,81]. In fact, the anchor residues typically bind strongly to the surface, while the remaining residues are either weakly bound or not bound in most configurations. In addition, given their reduced size, SBPs can experience structural confinement caused by intramolecular steric repulsions and misorientation of relevant side chains [95]. These observations lead to the hypothesis that non-binding residues in SBPs might allow for the optimal orientation of the anchor amino acids with respect to the surface.

The orientation of key residues is equally relevant in solid-binding proteins and solid-binding domains. For example, a rigid structure is necessary for the binding of some IBPs to ice crystals. These proteins feature precisely arranged one- or two-dimensional arrays of threonines [88]. These residues are all projected away from the ice-binding site with the same orientation, maximizing their possible interactions with matching crystal planes. Conversely, some solid-binding domains, like the silica-binding protein Si-tag, lack a stable three-dimensional structure and can remain partially or totally unfolded under physiological conditions [96]. These intrinsically disordered proteins can assume an optimal conformation upon binding with a solid surface, adapting their structure to maximize the intermolecular interface [[97], [98], [99]]. The majority of proteins involved in biomineralization appears to be intrinsically disordered, and, at least for this application, structural flexibility seems to be preferable to rigidity when interacting with solid surfaces [100].

Third, the binding strength is determined by the number of amino acids involved in surface-binding: changing the number of amino acids or solid-binding domains can be used to tune the strength of binding to the solid surface [101]. For example, the affinity of an SBP can be increased by repeating in tandem the sequences of interest. This approach was successfully applied to many inorganic surfaces [102,103], but satisfactory results are not always guaranteed [104,105]. In these cases, the connections between SBPs presumably introduced steric constraints, preventing optimal orientation of key residues for binding. Alternatively, multiple copies of an SBPs can be displayed on suitable molecular scaffolds to engineer multivalent binding [106]. This approach has been applied successfully in various cases, leading to dramatically enhanced affinities using both synthetic [[107], [108], [109]] and protein-based scaffold structures [110,111].

## 3 Biomedical applications of solid-binding peptides

In the following section, we will outline a selection of successful biomedical applications of solid-binding peptides and proteins, for solid surfaces such as calcium phosphate minerals, silicates, ice, metals, metal oxides, plastics, and graphene. All *in vivo* results were obtained in animal models, unless stated otherwise. A list of the solid-binding peptides and proteins discussed in this section can be found in Table 1.

**Table 1:** List of solid-binding peptides featured in this review, listed per surface in alphabetical order. We report the amino acid sequences of short peptides (<30 residues), while longer peptides and domains are reported with their original nomenclature.

Target	Sequence	Application	Reference
Gold	MHGKTQATSGTIQS	Theranostics, biosensing	[256,260,261,[265], [266], [267], [268], [269], [270]], first described in [50]
	WALRRSIRRQSY	Theranostics	[257], first described in [333]
	ASHQWAWKWE	Theranostics	[258], first described in [255]
	LKAHLPPSRLPS	Biosensing	[262], first described in [334]
	WAGAKRLVLRRE	Biosensing	[271], first described in [333]
	AYSSGAPPMPPF	Material synthesis	[259], first described in [278]
Graphene	HSSYWYAFNNKT	Biosensing	[327,329,330], first described in [335]
	IMVTESSDYSSY	Biosensing	[328]
	HNWYHWWPH	Biocompatibility	[331], first described in [336]
Hydroxyapatite	Poly(E)	Osteoinductivity, material synthesis, drug delivery	[[116], [117], [118], [119], [120], [121], [122], [123], [124], [125], [126], [127], [128], [129], [130],^153^,[155], [156], [157], [158],^164^,^171^,^174^,^178^]
	Poly(D)	Osteoinductivity, drug delivery	[[131], [132], [133], [134],[170], [171], [172], [173],^176^,^177^,[179], [180], [181]]
	γEPRRγEVAγEL	Osteoinductivity	[[136], [137], [138], [139], [140], [141]], first described in [337]
	VTKHLNQISQSY	Osteoinductivity	[[142], [143], [144], [145], [146]], first described in [338]
	TKREEVD	Remineralization	[148,168]
	SYENSHSQAINVDRT	Remineralization	[149]
	TKRQQVV	Remineralization	[150]
	DRNLGDSLHRQEI	Remineralization	[151]
	KYKQKRRSYK	Remineralization	[152]
	DpSpSEEK	Remineralization	[153]
	CMLPHHGAC	Multi-material synthesis, biocompatibility, imaging	[154,[160], [161], [162],^164^,^166^,^183^]
	MSESDSSDSDSKS	Multi-material synthesis	[159,163]
	NNHYLPR	Biocompatibility	[165], first described in [339]
	SVSVGMKPSPRP	Biocompatibility	[167]
	(DSS)_6_	Drug delivery	[175], first described in [340]
	STDKTKREEVD	Drug delivery	[182], first described in [341]
	KNFQSRSH	Imaging	[184]
Polycaprolactone	LCI peptide (47 ​amino acids)	Material synthesis	[309,320], first described in [342,343]
Poly (chloro-*p*-xylylene) (Parylene C)	LCI peptide	Material synthesis	[323]
	CecA peptide (37 ​amino acids)	Material synthesis	[323], first described in [344]
Polydimethylsiloxane (PDMS)	LCI peptide	Biocompatibility	[318,345]
Poly (methyl methacrylate) (PMMA)	ELWRPTR	Biocompatibility	[317], first described in [346]
Poly (4-methylpentene) (PMP)	LCI peptide	Multi-material synthesis	[321,322,345]
Polystyrene	RIIIRRIRR	Biocompatibility	[316], first described in [347]
	LCI peptide	Biocompatibility	[345]
Polyurethane acrylate (PUA)	AIRGIRGIRGIR	Biocompatibility	[319]
Silica	RGRRRRLSCRLL	Drug delivery	[211], first described in [348]
	SSKKSGSYSGSKGSKRRIL	Drug delivery, material synthesis, biosensing, biocompatibility	[[202], [203], [204],^206^,^209^,^212^,^215^,^219^,^221^,^222^,^232^,^233^,[236], [237], [238], [239]], first described in [349]
	L-linker (95 ​amino acids)	Drug delivery, biosensing	[213,214,[229], [230], [231]], first described in [350]
	RKKRKKRKKRKK	Drug delivery	[220], first described in [351,352]
	KPTHHHHHHDG	Material synthesis, drug delivery	[223], first described in [348]
	Si-tag (273 ​amino acids)	Biosensing,	[[226], [227], [228]], first described in [19]
	SSRSSSHRRHDHHDHRRGS	Biosensing, material synthesis, biocompatibility	[207,233], first described in [353]
	DSARGFKKPGKR	Biosensing	[234], first described in [354]
	MSPHPHPRHHHT	Biocompatibility	[208,209], first described in [348]
Silver	NPSSLFRYLPSD	Nanoparticle synthesis, material synthesis	[275,285], first described in [278]
	Ac-(3′-PyA)LRLRLRL (3′-PyA)-CONH_2_	Material synthesis	[276]
	AYSSGAPPMPPF	Nanoparticle synthesis	[286], first described in [278]
	EQLGVRKELRGV	Biocompatibility, material synthesis	[287,288,290], first described in [355]
	WSWRSPTPHVVT	Antimicrobial material synthesis	[289], first described in [277]
Titanium	DSSEE	Biocompatibility	[293], first described in [356]
	RPRENRGRERGL	Biocompatibility	[294,295,[304], [305], [306], [307]]
	SRPNGYGGSESS	Biocompatibility	[294,304,305]
	CGHTHYHAVRTQT	Biocompatibility	[297], first described in [352]
	DYFSSPYYEQLF	Biocompatibility	[298], first described in [110,357]
	RKLPDA	Biocompatibility	[296,[299], [300], [301]], first described in [57]
	RKLPDAPGMHTW	Biocompatibility	[302,303], first described in [57]
	HAYKQPVLSTPF	Biocompatibility	[304]

### 3.1 Calcium phosphate minerals

Calcium phosphate (CaP) minerals are the main inorganic components of human bones and teeth. Hydroxyapatite (HA) crystals are the prevalent mineral constituent found in native bone, while phosphate and calcium ions are used for synthetic bone replacements such as β-tricalcium phosphate (β-TCP). Calcium phosphates are therefore highly biocompatible and osteoconductive, making them ideal biomaterials for regenerative therapies, as bone fillers or as coatings for metal implants [112]. Calcium phosphates present good mechanical and physical properties, but they lack the ability to recruit immature cells and induce their differentiation (osteoinductivity). For this reason, considerable research efforts have been dedicated to the functionalization of CaP-based materials with therapeutic growth factors.

Since many proteins and peptides are involved in the formation of hard tissues, SBPs are a popular choice for the immobilization of bioactive molecules on CaP surfaces. A particularly attractive feature of SBP-mediated functionalization is the reversibility and tunability of the binding strength. Localized delivery and sustained release of growth factors, for example, can effectively mimic the physiological chemotactic gradient required for tissue growth [113,114]. Furthermore, HA surfaces include only few functional groups for covalent bonding, making adsorption-based methods an attractive option [115]. In the current section, we will discuss the application of CaP-binding peptides in implant coatings and remineralization therapies, material synthesis, drug delivery, and vaccine development.

The extracellular matrix of bone is rich in acidic non-collagenous proteins, involved in cell migration and differentiation [89]. Many of these proteins have been shown to bind to HA crystals and to regulate bone formation. Unsurprisingly, the simplest HA-binding peptides consist of repetitive acidic amino acids. One of the first successful attempts at cell attachment onto HA using acidic binding peptides was reported by Fujizawa et al., featuring a poly-glutamate heptapeptide (E7) for HA binding [116]. The fusion protein also contained an RGD peptide for cell binding, resulting in improved *in vitro* osteoblast attachment and mineralization [117]. The same HA-binding peptide was then adopted in similar designs with the three other cell-binding peptides, showing that not all cell-binding motifs elicit the desired response [118,119]. Promising results were reported for fusions with the collagen-derived motif DGEA: the peptide coating was stable *in vivo* for at least 2 months when used on HA, allograft bone, β-TCP, and Ca-containing cement [120,121]. Interestingly, the authors showed that varying the length of the poly-glutamate peptide is an effective method to tune the release rate of the adsorbed biomolecules [118,120].

In the same series of experiments, Culpepper et al. showed similar results when using a peptide derived from the bone morphogenic protein 2 (BMP-2) fused to the HA-binding peptide E7 [121]. The same modular protein was extensively researched in subsequent studies, proving its remarkable osteoinductivity both *in vitro* and *in vivo* on allograft bone, bone scaffolds, bone substitutes, mineralized silk fibres, and hybrid aerogels [[122], [123], [124], [125], [126]]. Tests performed with different osteogenic peptide motifs yielded similarly positive results [127]. For example, a VEGF-derived peptide fused to the HA-binding peptide E7 could be slowly released from AH disks to stimulate neovascularization *in vitro* [128,129]. Finally, the poly-glutamate octapeptide E8 was reported to effectively anchor recombinant BMP2 to HA disks [130].

More recently, the poly-aspartate heptapeptide D7 was investigated in combination with a BMP-2-derived peptide. The fusion protein showed higher affinity for HA-surfaces compared to the E7-labeled peptide, exerting excellent bone regeneration capabilities *in vitro* and *in vivo* on synthetic bone scaffolds [[131], [132], [133]]. Shorter poly(D) peptides have been successfully applied for the sustained release of osteogenic hormones [134].

Non-repetitive HA-binding peptides have also been extensively studied for cell adhesion and bone factor delivery strategies [135]. For example, an osteocalcin-derived HA-binding peptide was fused to a BMP-2-derived peptide [136]. Follow up studies revealed that the fusion protein could target bone tissues, while promoting stem cell differentiation *in vitro* and bone healing *in vivo* on HA-coated surfaces [[136], [137], [138], [139]]. A fusion protein with a VEGF-derived peptide was tested *in vitro* on HA and β-TCP surfaces, confirming the ability to induce endothelial cell proliferation [140,141].

Ramaraju et al. reported the use of an HA-binding peptide derived from phage display [142]. Interestingly, the peptide was shown to inhibit cell mineralization *in vitro*, possibly by sequestering calcium ions and delaying their deposition [143]. Nonetheless, when the peptide was fused to other cell-binding domains, the resulting protein could effectively coat 2D and 3D mineralized scaffolds and induce osteogenesis both *in vitro* and *in vivo* [[144], [145], [146]]. In these instances, the phosphorylation of the serines in the HA-binding peptide has been related to an increase in its binding strength [147]. Consequently, a lack of phosphorylation resulted in weaker binding without adverse effects. These results highlight the need for a detailed understanding the binding mechanisms of SBPs to better tailor their usage.

HA-binding peptides have found several applications in the design of tooth remineralization therapies. Peptides used for these applications are usually derived from the enamel matrix, a protein-rich mixture that forms the hard and highly mineralized tooth tissue. In a few recent examples, remineralization of damaged teeth was achieved both *in vivo* and *in vitro* thanks to four HA-binding peptides derived from the enamel matrix proteins amelogenin and tuftelin [[148], [149], [150], [151]]. Positive results were also achieved with acidic peptides derived from the dental epithelium protein copine 7 or the salivary protein statherin [152,153]. Notably, three of these HA-binding peptides improved remineralization without being fused to other functional peptides. This finding indicates that these HA-binding peptides can exert a physiological function without being fused to another functional domain.

The interactions of SBPs and CaP minerals have found limited but noteworthy applications in the improvement of CaP-based biomaterials [154]. A straightforward yet effective approach involved the synthesis of a protein-mineral nanocomposite. The poly-glutamate octapeptide E8 was fused to charged elastin-like polypeptides (ELPs) and mixed with calcium-based fillers [[155], [156], [157]]. The resulting materials showed increased stability in fluids and improved binding to teeth, without impacting their biocompatibility *in vitro* [156,158]. Other proof-of-principle examples relied on more complex natural or synthetic polymer materials such as spider silk fibres, polyetherimide (PEI) films, methacrylate antibacterial adhesives, and shape memory polymer gels [[159], [160], [161], [162]]. Finally, a notable example was recently reported by Lauria and co-authors [163,164]. Potato virus X nanoparticles were engineered to expose both cell-binding and HA-binding peptide on their surface, then subsequently loaded into hydrogels, mimicking the extracellular matrix. Remarkably, the viral nanoparticles initiated mineralization inside the hydrogel and sustained osteogenesis *in vitro*, showing good potential as an innovative composite for bone tissue engineering.

Next, various proof-of-concept studies have addressed the use of HA-binding peptides for the immobilization of antimicrobial peptides (AMPs) against oral pathogens. In most instances, a HA-binding peptide was fused to an AMP, directly or via a short flexible linker. Huang et al. showed how the fusion protein could effectively disrupt *S. mutans* biofilms *in vitro*, although less effectively than conventional antibiotics [165]. The fusion protein could retain cytocompatibility and stability in human saliva. Comparable results were obtained with similar designs against *Escherichia coli* [166,167]. Finally, using an amelogenin-derived HA-binding peptide fused to an AMP is an effective strategy to achieve both antimicrobial activity and carious tissue remineralization [168].

Bone-specific drug delivery via HA-binding peptides has also been explored in the past decade. Effective delivery of drugs to bone tissues is complicated by the limited blood supply and the bone marrow-blood barrier [169]. Nonetheless, bones are a unique solid material within the human body and peptide-mediated delivery has proved effective and with limited off-target effects. In a first proof-of-concept, a repetitive acidic oligopeptide (poly(E)) was directly tethered to an estrogen and tested *in vivo* as possible therapy against osteoporosis [170]. These promising results stimulated the testing of other osteotropic drugs fused to acidic HA-binding tags [171,172]. Repetitive acidic tags were successfully used for the targeted delivery of enzymes, lyposomes, and nanoparticles to bone tissue, showing in all cases highly promising results both *in vitro* and *in vivo* in animal models [[173], [174], [175], [176], [177], [178], [179]]. Poly(D) peptides have also been applied in gene delivery therapies targeted to bone tissue. For example, the adeno-associated virus 2 vector used for gene delivery was modified to expose HA-binding peptides, with clear improvements [180,181]. Finally, Ren et al. reported an elegant strategy for the delivery of drugs to specific cells in the bone tissue: the authors combined an HA-binding peptide derived from amelogenin and an osteoblast-specific aptamer to develop a nanocarrier with improved specificity both *in vitro* and *in vivo* [182].

Targeted delivery to HA surfaces furthermore finds promising applications in the field of bioimaging, especially for *in vivo* bone imaging, although only proof-of-concept studies have so far been reported. In a first attempt, an HA-binding peptide derived from phage display was fused to a green fluorescent protein derivative [183]. The resulting fusion protein was tested *in vitro* on acellular cementum of human teeth, giving positive results with fluorescence microscopy. In another example, Bang et al. identified a supposedly highly specific HA-binding peptide via phage display and combined it with the fluorescent Cy5.5 dye [184]. During *in vivo* real-time whole-body imaging, the conjugate showed preferential binding for bone tissues. The development of probes for nuclear imaging was also attempted, but with unsatisfactory results [185].

**Fig. 4:**
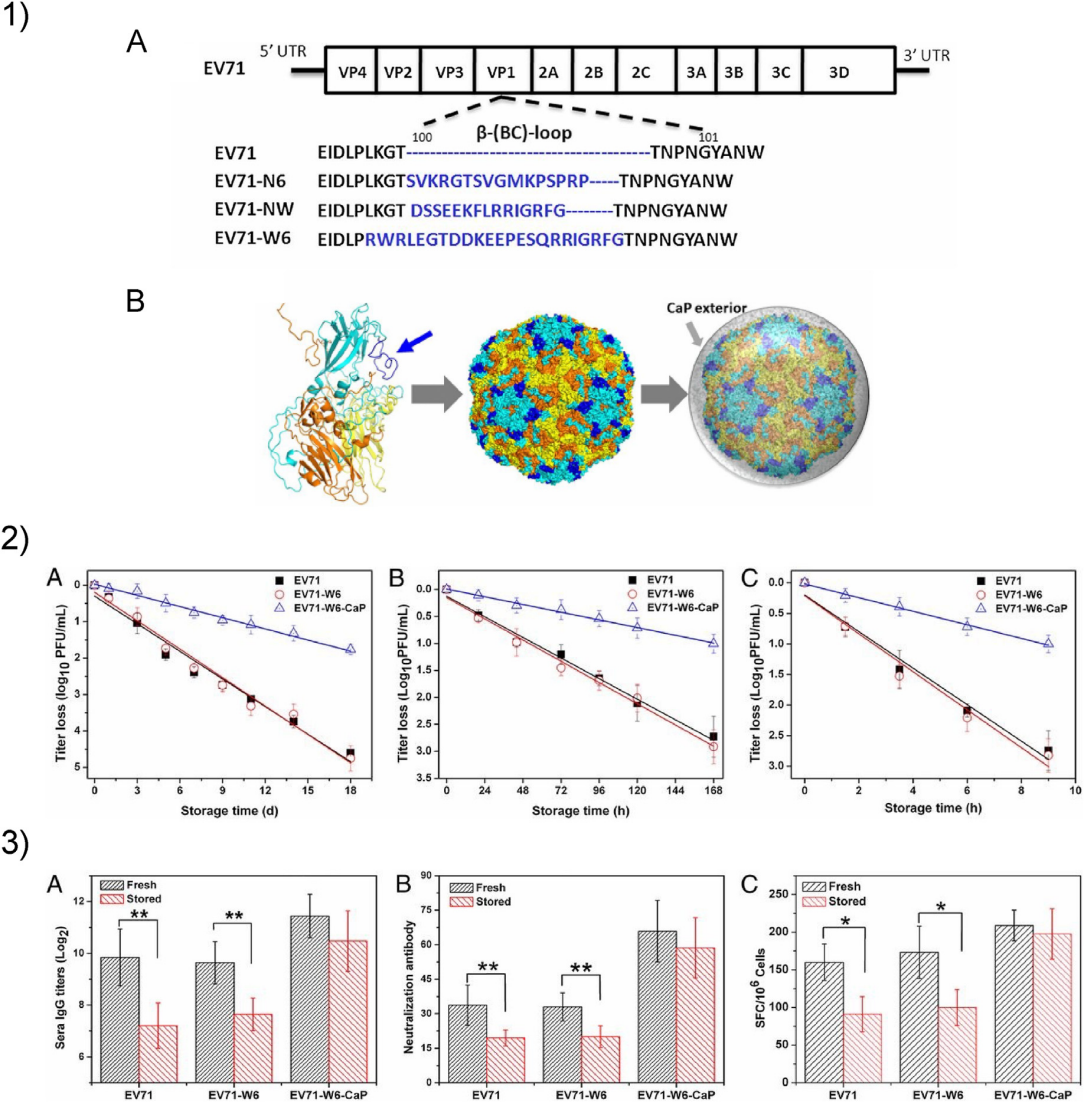
CaP-binding peptides can be used in the design of biomineralized vaccine carriers. 1) The CaP-binding sequence was inserted in a capsid protein of human enterovirus type 71 (EV71) (A) to achieve a uniform distribution on the virion surface (dark blue) and induce *in situ* mineralization (B). 2) *In vitro* test of virus thermostability at 26 ​°C ​(A), 37 ​°C ​(B), and 42 ​°C ​(C). After incubation, the infectivity was evaluated by plaque assay. The percentage of infectivity is shown in logarithmic scale as a function of storage time. 3) Testing of vaccines, either fresh or stored at 37 ​°C for 5 days, on animal models. EV71-specific IgG titres (A) and neutralizing antibodies (B) induced by uncoated (EV71 and EV71-W6) and CaP-coated viral particles (EV71-W6-CaP), 4 weeks after immunization. (C) Frequency of EV71-specific splenocytes in immunized mice 2 weeks. Adapted from Ref. [186].

Lastly, CaP minerals have found applications in vaccine engineering. For example, biomimetic mineralization of viral particles was proven to increase their efficacy as vaccine vectors. CaP mineral shells have been shown to provide physical and chemical protection to the viral particle, resulting in enhanced infectivity, circumvention of neutralizing antibodies, avoidance of pre-existing immunity, and improved thermal stability [[186], [187], [188], [189]]. CaP-binding peptides have therefore found an application in this field. A relevant example of biomineralized vaccine vectors is reported in Fig. 4. In this study, viral particles have been engineered to expose CaP-binding peptides on their surface (Fig. 4.1) [190]. The resulting mineralized particles showed increased thermostability compared to uncoated particles, while improving their ability to induce an immune response (Figs. 4.2–3). Furthermore, CaP nanoparticles have been successfully administered as biocompatible vaccine adjuvants [191]. In this regard, CaP-binding peptides can both guide the synthesis of the nanoparticles and allow the exposition of antigens on their outer shell [192,193].

In conclusion, CaP-binding peptides have been extensively applied to a vast range of biomedical applications, often yielding promising results in animal model experiments. This success lies in the flexibility of peptide-based functionalization strategies, given the reversibility and tunability of binding and the inherent biocompatibility. It is also worth noting that peptides derived from extracellular matrix proteins have provided the most promising results.

### 3.2 Silicates

Silica is the most abundant biomineral found in the Earth's lithosphere, making up almost a third of its crust [194]. Given the abundancy of this material, it is not surprising that silica mineralization is extensively used by many organisms, such as diatoms and sponges, for the creation of complex extracellular structures. The unique properties of silica make it an ideal material for biotechnological and biomedical applications. Not only are silica-based materials generally biocompatible and non-toxic, they also present excellent thermal and pH stability, chemical inertness, and mechanical resistance [195]. For these reasons, silica is widely used for the fabrication of implantable devices, nanoparticle-mediated drug delivery, biosensing, and bioencapsulation strategies [196]. Although silica surfaces present moieties for covalent modification, there is growing interest in simpler adsorption-based strategies. Fortunately, the increasing understanding of biological silicification has made silica-binding peptides an attractive alternative for the modification of silica surfaces. For example, peptides derived from natural proteins such as silaffin R5 have been shown to mediate the formation of nanoparticles *in vitro*, allowing for the facile immobilization of biomolecules on newly synthesized particles [[197], [198], [199]]. In general, peptides enriched in positively charged residues, arginine in particular, have been shown to preferentially bind to silica surfaces [92]. This finding suggests that electrostatic interactions are a major contributor to the binding affinity on negatively charged silica surfaces. In this section, we will provide an overview of suggested biomedical applications of silica-binding peptides as part of coatings for implants, for drug delivery, and in biosensing.

Silicon and silicate ions have a direct influence on the formation and repair of bone tissue, and in recent years, bioactive glasses have been extensively evaluated as possible bone graft materials [200,201]. Silica-binding peptides have therefore been used for the synthesis and functionalization of silica-based biomaterials, such as biomimetic silk films and hydrogel layers [[202], [203], [204]]. Guo et al. reported the use of a peptide solution as ink for the inkjet printing of silica micropatterns on a hydrogel [205]. The printed silica-binding peptide mediated the silicification of the micropatterns and allowed the alignment of mesenchymal stem cells on the surface *in vitro*. In a different approach, a bifunctional peptide was used for the multi-layer silicification of titanium implantable surfaces, resulting in improved bone formation *in vitro* and *in vivo* [206]. Finally, Yun et al. prepared a porous biomineral composite material with the help of a bifunctional peptide and marine phytoplankton [207]. The porous calcium carbonate shell of coccolithophores (coccolith) was mixed with the peptide solution, resulting in a silicified material with high *in vitro* cytocompatibility and osteoinductivity *in vivo*.

The antibacterial activity of biomaterial surfaces is an important functionality of implantable materials. Implantable silica surfaces have been modified to reduce the chance of bacterial colonization. In two recent publications, two bacteriolytic enzymes were adsorbed on silica and silicone surfaces via silica-binding peptides [208,209]. In one case, the coating showed good antibacterial properties against *Staphylococcus aureus* both in static and dynamic biofilms, providing a proof-of-concept for its usage in clinically relevant applications [209]. Interestingly, antimicrobial peptides (AMPs) were not successfully included in similar bifunctional designs, to the authors’ knowledge. We speculate that the cationic nature of most AMPs might compete with the silica-binding peptides for the adhesion to negatively charged silica surfaces, resulting in reduced and misoriented peptide immobilization. Interestingly, the positive charge of AMPs can be advantageously used for the direct adsorption on mesoporous silica particles, providing sufficient adsorption strength for delivery, release and functionality [210].

The targeted delivery of bioactive molecules has extensively relied on silica nanoparticles, both porous and non-porous, as inert and biocompatible carriers. In this context, silica-binding peptides have found many applications. For example, Lu et al. reported the synthesis of a bi-functional peptide binding both silica and RNA. The peptide was used for the nanoparticle-mediated delivery of miRNA, resulting in effective gene silencing in vitro cell cultures [211]. More recently, a silica-binding peptide linked to bioactive polyphenols was used for the functionalization of silica nanoparticles and the delivery to the nucleus of cancerous cells *in vitro* [212]. Silica nanoparticles coated via silica-binding peptides have also been used for cell targeting. For example, a silica-binding peptide was fused to an antibody-binding domain, providing a flexible platform for the conjugation of different IgG antibodies (Abs) to the surface of nanoparticles for photodynamic therapy and cancerous cell targeting [213,214].

Silica-binding peptides have been shown to induce local growth of silica. For this reason, they have been used to explore alternative methods for drug delivery, like particle encapsulation to increase the loading of poorly soluble drugs. Since silicification was proved to increase the thermal and chemical stability of fluorescent proteins, enzymes, viruses, and eukaryotic cells, this approach was tested with therapeutical compounds [[215], [216], [217], [218]]. For example, the use of a bifunctional polypeptide allowed to stabilize a model hydrophobic drug into polypeptide assemblies and induce the formation of a silica shell around the polypeptide/drug core, with positive results both *in vitro* and *in vivo* [219,220]. More recently, other molecular scaffolds were combined with silicification. After showing that the transport protein ferritin can be modified with a silica-binding peptide to create a silica-enveloped drug carrier, Ki et al. proved that a second drug can be loaded into the outer silica shell [221,222]. The resulting dual delivery system was tested *in vitro*, revealing that the two drugs can be released at different rates after cellular intake. Modified ferritin was also used for the controlled synthesis of silica nanoparticles with an encapsuled anticancer drug [223]. Finally, an elegant delivery method was proposed by Delalat et al., via live diatom silica immobilization [224]. In this method, diatoms are genetically modified with a synthetic gene encoding the protein of interest fused to silaffin-derived peptides. Since these peptides are a natural component of diatom biosilica, they can direct the permanent immobilization of the protein of interest on the newly formed extracellular surface. In this way, the authors could functionalize diatom biosilica with an antibody-binding peptide. The antibody-labeled biosilica was then loaded with micelles containing an anticancer drug and successfully tested both *in vitro* and on animal models.

Silica is an important material for surface-bound biosensing applications, either as a macroscopic surface or in the form of silica micro- and nanoparticles [225]. Since surface modification is a key aspect in any biosensor design, silica-binding peptides have provided a valuable tool for the oriented immobilization of bioactive detection elements. In a first proof-of-principle experiment, antibodies were immobilized on silica surfaces via a fusion peptide and used for the detection of allergens on an optical ring resonators device [[226], [227], [228]]. A similar approach was explored for the detection of pathogens, both on flat surfaces and on silica nanoparticles [[229], [230], [231]]. Silica-binding peptides were also tested for the bioencapsulation of enzymes for the detection of glucose, for immunoprecipitation essays, but also for the detection of antibiotics and the detection of asbestos in live cells *in vitro* [[232], [233], [234], [235]].

**Fig. 5:**
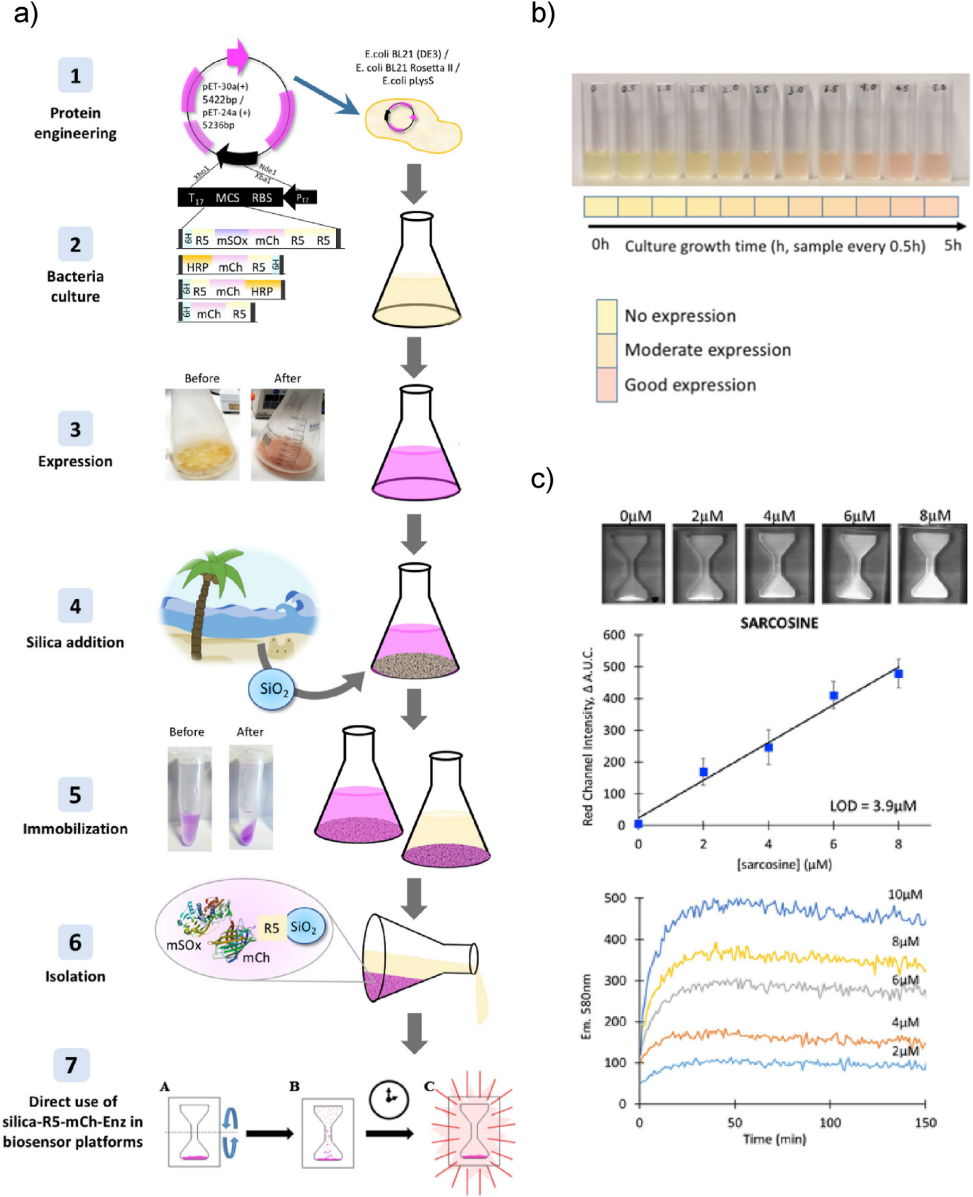
Silica-binding peptides are used for the immobilization of protein on silica particles in sarcosine detection biosensor. a) Schematic illustration of production and purification steps in the “gene to device” strategy. b) Visual change in colour of induced bacterial cultures allows semi-quantitative detection of protein expression levels with the help of a colour card. c) Hourglass biosensing device can detect sarcosine in solution, providing a quantitative output. The device can be reliably used for end-point essays. Adapted from Refs. [236,238], with permission from Elsevier.

An elegant series of recent publications has highlighted how silica-binding peptides can be adapted for the design of cheap biosensing platforms. Henderson et al. designed a fusion protein featuring a silica-binding peptide, the fluorescent protein mCherry and the enzyme sarcosine oxidase (SOx) [236]. The biosensing system is illustrated ib Fig. 5. In their design, the silica-binding peptide functions as immobilization and purification tag, the mCherry marker provides a visual reference for the monitoring of protein production, while SOx is used to generate H_2_O_2_ in the presence of the prostate cancer biomarker sarcosine (Fig. 5a–b). A second fusion protein, containing the enzyme horseradish peroxidase (HRP) instead of SOx, can convert the H_2_O_2_ produced by SOx into a fluorescent product and ultimately allow the colorimetric detection of sarcosine [237]. Silica particles extracted from sand have been employed for the purification of the recombinant protein directly from bacterial crude extract. The functionalized particles were then inserted in a flat, hourglass shaped device (Fig. 5c). The device was used for the detection of sarcosine by inverting the hourglass, allowing the particles to sediment and come into contact with the whole sample volume [238]. This “falling particle” biosensor was recently adapted for the DNA polymerase-based clinical diagnosis of malaria infections in Ghana, proving how solid-binding peptides are invaluable tools for the design of cheap, simple and effective biosensing devices [239].

In conclusion, silica-binding peptides are versatile tools for many *in vitro* applications, from drug delivery to biosensing. Given the natural abundance and cheapness of silicates, peptide-based surface functionalization will likely become increasingly relevant in the synthesis of silicon-based biomaterials. Furthermore, given the importance of silicates in bone remineralization, *in vivo* applications might be explored more extensively.

### 3.3 Ice crystals

While liquid water is fundamental for the survival of any organism, the formation of ice crystals can have lethal consequences for cells and tissues. Life at sub-zero temperatures is made possible by cryoprotective molecules such as ice-binding proteins (IBPs), which bind ice crystals and modulate crystal growth [20]. As mentioned in the introduction, the natural variety of IBP structures and sequences seems to demonstrate that there is no preferred way to bind a crystalline solid surface. Both disordered, glycosylated peptides and highly ordered proteins with molecularly flat binding interfaces are effective IBPs.

Since their discovery in 1969, IBPs have drawn substantial attention for their potential application in agriculture, food technology and biomedicine [240]. A particularly promising biomedical application is the cryopreservation of cells, embryos, organs, and other tissues. While widespread research on IBPs and IBP analogues is still ongoing, it should be emphasized that research on the use of IBPs in cryopreservation is still in its early stages. Considering the wealth of excellent and comprehensive reviews on the topic, we refer the reader to recent general reviews on cryopreservation [241], the possible role of IBPs in cryopreservation in general [21,242,243], and their use in the preservation of organs and tissues [244] and in reproductive medicine in particular [245].

### 3.4 Metals and metal oxides

Metals have played a paramount role in the history of medicine since ancient times: the Greek physician Hippocrates employed silver for the treatment of wounds, while gold was already used in ancient China 4 millennia ago. In the last decades, metals have found new biomedical applications especially in the field of nanomedicine, due to their unique physical and chemical properties [246]. Next to gold and silver, titanium is a key metal used as biomaterial. In the following sections, we will focus on biomedical applications of gold-, silver-, and titanium/titanium oxide-binding peptides respectively. In the case of gold and silver, we will mainly review therapeutic and biosensing applications, with a focus on nanoparticle functionalization. In the case of titanium, we will instead review the application of titanium-binding peptides in coatings for implants.

Among the noble metals, gold (Au) has found practical applications in medical treatments [247,248]. For example, one of the clinical uses of gold dates back to the late 1920s, in the form of gold salts for the treatment of rheumatoid arthritis [249]. In the past decades, gold nanoparticles (AuNPs or GNPs) and gold nanoclusters (AuNCs) have been extensively researched, showing a vast range of potential biomedical applications [[250], [251], [252], [253]]. The success of gold nanostructures is due to their unique physical and chemical properties, including biocompatibility, bioinertness, tuneable morphologies and optical properties, and resistance to corrosion and oxidation [250]. Furthermore, since AuNPs present a simple surface chemistry, various conjugation strategies have been developed for their functionalization. Many synthesis and functionalization strategies rely on the covalent attachment of bioactive molecules, which grants chemical stability at the expense of flexibility of use. For this reason, gold-binding peptides have been explored for the facile and efficient functionalization of AuNPs, both in therapeutic and biosensing applications. In the case of Au-binding peptides, various amino acids have been reported to recognize the Au lattice: aromatic amino acids (Y, F, W), basic amino acids (R, K), and polar hydroxyl-containing amino acids (S, Y) appear to have a particularly high affinity for the gold-water interface [254]. Expectedly, many Au-binding peptides have been isolated in recent years [255]. It should also be remembered that cysteine residues (C) covalently bind to gold surfaces via their thiol side group.

Au-binding peptides have found limited but noteworthy applications in photothermal therapy. For example, Oh et al. designed a modified T7 phage displaying both an Au-binding peptide and a prostate cancer cell-binding peptide [256]. The modified phages could assemble a thin AuNP shell and act as nanocarriers for cancer-targeted photothermal therapy *in vitro*. Similar results were achieved more recently with nuclei-targeting AuNPs by Gao et al. [257] In their article, the authors speculate that the aggregation of AuNPs in the cancer cell nucleus could produce more heat during photothermal therapy, resulting in a reduced cell viability. In another recent example, Tanaka et al. synthesized triangular Au nanoplates using a Au-binding peptide, and successfully employed them in vitro photothermal therapy [258]. Furthermore, the recent report of the intracellular synthesis of AuNPs thanks to a fusion peptide shows promising future applications in nanomaterial synthesis and, possibly, theranostics and bioimaging [259].

**Fig. 6:**
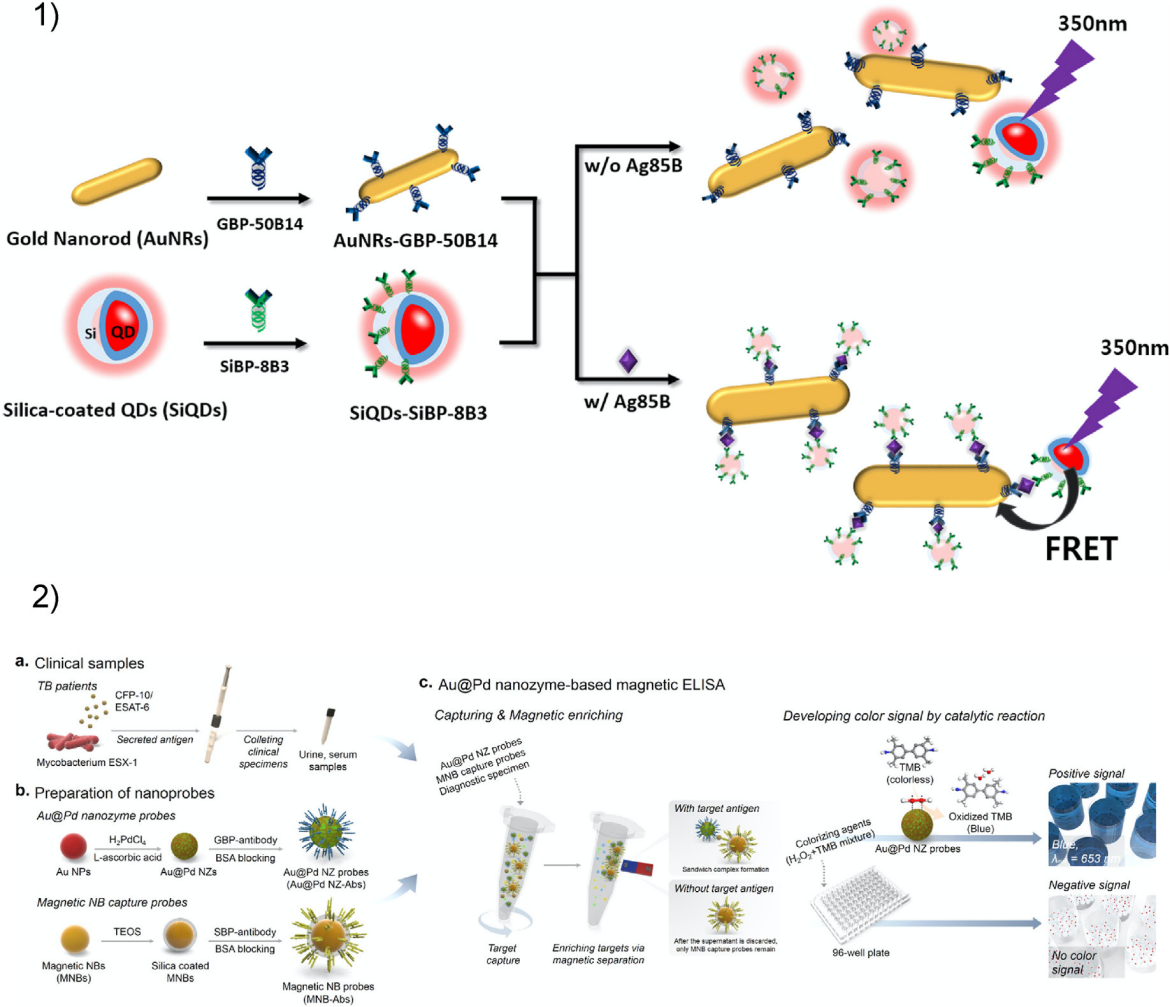
1) Schematic illustration of FRET-based sandwich assay for the detection of a secreted *M. tuberculosis* antigen. Gold nanorods (AuNRs) are functionalized with 50B14 antibodies fused to a gold-binding peptide. Silica-coated quantum dots (SiQDs) are similarly coated via a silica-binding peptide. In the absence of antigen, a fluorescence signal is recorded. When the antigen Ag85 ​B is present, the AuNRs and SiQDs form an immunocomplex that quenches the fluorescent signal of the SiQDs thanks to FRET. Reprinted from Ref. [272], with permission from Elsevier. 2) Schematic illustration of nanozyme-based magnetic ELISA assay for the detection of a secreted *M. tuberculosis* antigen. After the preparation of the clinical samples (a), two nanoprobes are synthesized and modified (b). Bimetallic (Au/Pd) nanoparticles are functionalized with G2 antibody fused to a gold-binding peptide, while magnetic silica nanoparticles are similarly coated via a silica-binding peptide. The two nanoprobes are then mixed with the clinical samples and captured via magnetic separation (c). In the presence of the bacterial antigen, the two nanoprobes form a sandwich immunocomplex that can be detected thanks to the peroxidase-like activity of the bimetallic nanoparticle. If the antigen is not detected, only the magnetic silica nanoparticles are separated, and no signal is recorded. Reprinted with permission from Ref. [273]. Copyright 2021 American Chemical Society.

Gold is a key material in the biosensing field, in both plasmon-based sensing and electrochemical sensing. In biosensing platforms, the detection of analytes relies on the precise, homogeneous and oriented immobilization of a biological sensitive element. In this regard, Au-binding peptides provide a simple and fast method for the functionalization of gold surfaces. For example, an Au-binding peptide has been successfully implemented in many electrochemical biosensors [260,261]. In a recent publication, Lee et al. fabricated an enzyme-based electrochemical sensor by fusing an Au-binding peptide to a glucose dehydrogenase [262]. The fusion protein was then adsorbed on a gold electrode surface and used for the detection and quantification of glucose in whole blood samples. The sensor showed high sensitivity and could be stored for over a month. It is important to remark that the fusion site (*C*-terminal or *N*-terminal) and the amino acid sequence of the Au-binding peptides are crucial parameters in the design of direct electron transfer-based biosensors. Both factors directly influence the orientation, the proximity and ultimately the interface between the enzyme and the electrode surface [263,264]. Other explored biosensor designs include ELISA-based approaches, optical biosensors or lab-on-a-chip microfluidic biosensors [[265], [266], [267], [268], [269], [270]]. Au-binding peptides have also been tethered to DNA for the detection of nucleic acid targets [271]. Finally, two representative biosensor designs are shown in Fig. 6. In their recent reports, Kim and co-authors described the use of an Au-binding peptide in a biosensor based on fluorescence resonance energy transfer (FRET) (Fig. 6.1) and a colorimetric biosensor for the detection of *Mycobacterium tuberculosis* (Fig. 6.2). Both of these examples highlight how both Au-binding peptides and silica-binding peptides can be included in biosensor designs with different rationales [272,273].

Similarly to gold, the medical properties of silver (Ag) are long known: starting from 1500 BCE, silver-containing compounds have been used as wide-spectrum antimicrobials for topical applications [274]. Silver ions appear to interfere with several intracellular processes and bind the negatively charged bacterial cell wall, increasing its permeability [274]. Nowadays, silver is generating an increasing interest as an alternative to conventional antibiotics, and the adsorption of silver ions onto medical devices is one of the proposed strategies in the fight against antibiotic resistance [274]. In this context, silver-binding peptides have provided help in the synthesis of silver-containing hybrid materials such as silk-silver fibres and antimicrobial hydrogels [275,276].

The number of Ag-binding peptides is low compared to other SBPs. In fact, only few combinatorial studies have attempted to identify Ag-binding sequences [[277], [278], [279]]. The systematic characterization of Ag-binding is less developed compared to other metals, both in experimental quantification and in molecular simulations [46,280]. Given the structural and physical similarities between silver and gold surfaces, however, the materials selectivity of SBPs has been investigated for these two metals. Interestingly, peptides initially selected for Ag surfaces could bind Au surfaces and vice versa, showing comparable binding affinities [23,281,282]. These results highlight how combinatorial techniques alone might be insufficient to select materials-specific sequences. Despite recent advancements, achieving preferential binding to Ag surfaces in the presence of gold still represents a substantial obstacle [46].

Silver nanoparticles (AgNPs) have gained popularity for cosmetics, surgical coatings, medical implants, and as bactericidal compounds, due to their large surface to volume ratio, small size, and chemical stability [283]. Since small AgNPs show the highest antimicrobial effect, silver-binding peptides have been used for the size-constrained synthesis of homogeneous AgNP populations [284,285]. The resulting AgNPs showed increased cytotoxicity against *E. coli*, *Pseudomonas aeruginosa*, *Salmonella typhimurium*, *Shigella flexneri* and *Bacillus subtilis* compared to chemically synthesized AgNPs. Bifunctional peptides have been exploited to facilitate the synthesis of AgNPs and reduce their cytotoxicity towards fibroblast cells [286,287]. The growth of bacterial biofilm can be effectively tackled by biofunctionalized AgNPs. Chen et al. fused a Ag-binding peptide with the enzyme dispersin B and adsorbed the protein on silver nanoparticles [288]. After the treatment, AgNPs could dissolve *S. epidermidis* biofilm and kill the released bacterial cells. Further efforts have been taken to make AgNPs pathogen selective. Kim et al., for example, tested two multifunctional proteins featuring a Ag-binding peptide and two cell wall binding domains [289]. The two proteins were adsorbed on AgNPs and subsequently incubated with mixtures of either *B. subtilis* and *Bacillus anthracis*, or *S. aureus* and *B. anthracis*. This approach resulted in the selective decontamination of only one pathogenic species from each mixture, proving the specificity of coated AgNPs in pathogen detection and killing.

Yet another application for Ag-binding peptides was recently explored by Woolfolk et al. The authors reported the synthesis of a bifunctional peptide for the coating of silver diamine fluoride (SDF)-treated tooth tissue [290]. SDF is applied to effectively slow the progression of dental caries. In an *in vitro* experiment, a Ag-binding peptide fused to a remineralization-inducing peptide promoted the remineralization of the affected area.

Titanium (Ti) and titanium alloys are the standard inert materials for the manufacturing of dental and orthopedic implants. The mechanical properties of titanium, alongside its biocompatibility and resistance to corrosion, make it ideal for implantable devices [291]. The lack of recognizable moieties on the implant, however, does not allow for the regulation of bone cell processes and can induce a foreign body reaction to the implant. For this reason, SBPs have been suggested as an easy and efficient approach for the functionalization of Ti surfaces to modulate the interactions of the implant with the surrounding tissue. The interfacial interactions of Ti and TiO_2_ in aqueous solutions and amino acids or peptides have hardly been investigated in detail [292] However, recent studies have highlighted the role of charged and aromatic amino acids in the binding affinity of Ti-binding peptides [86,292].

**Fig. 7:**
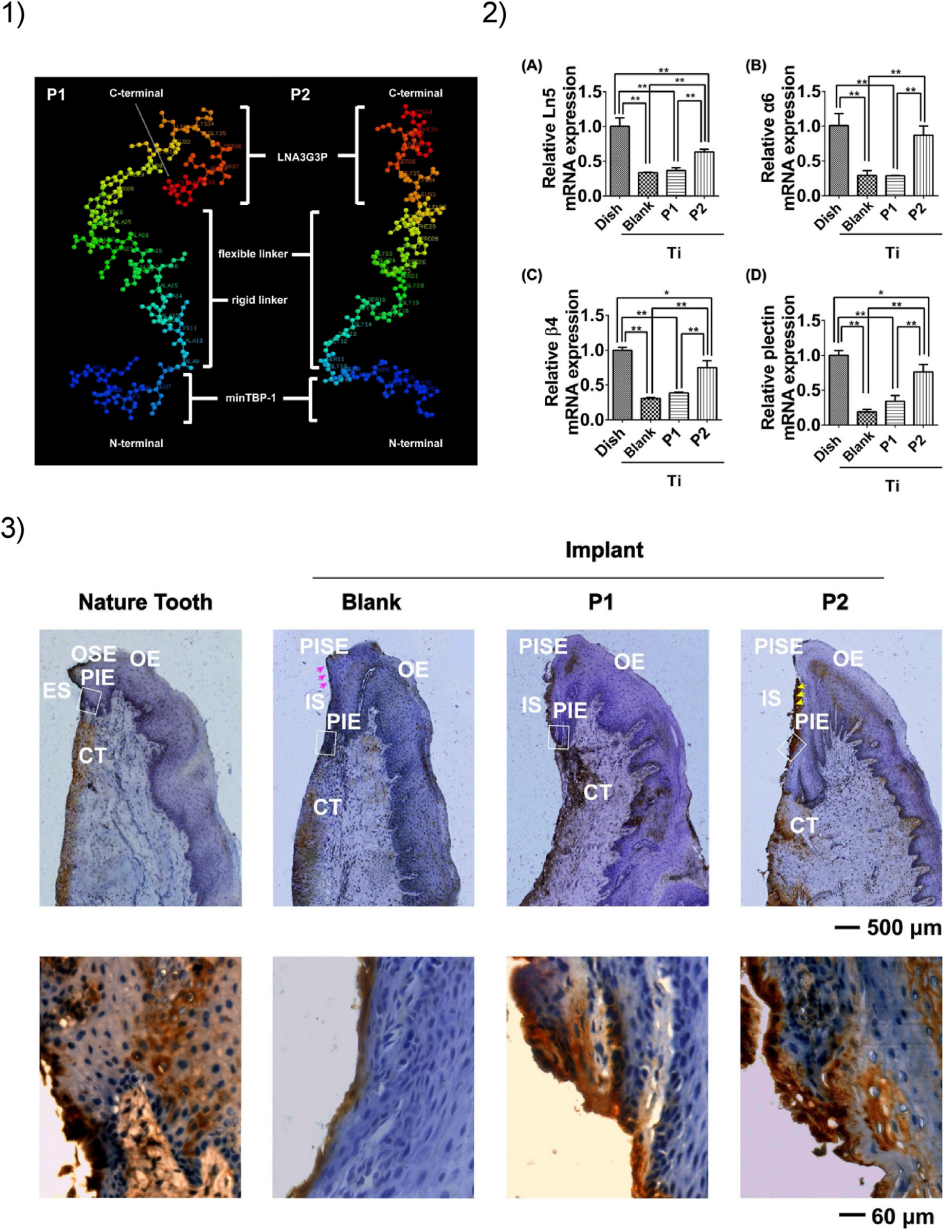
Example of a Ti-binding peptide used for the functionalization of dental titanium implants. 1) Pseudo-3D representation of fusion peptides P1 and P2. While the blue fragment at the *N*-terminal bins to the Ti surface, the peptide at the *C*-terminal binds to oral epithelial cells and activates soft tissue sealing around the implant. 2) Relative mRNA expression levels of *Ln-5*, *Integrin α6*, *Integrin β4*, and *Plectin* genes in human oral epithelial cells. The cells were cultured for 10 days on a culturing dish, uncoated titanium surfaces (bare) and peptide-coated titanium surfaces (P1 and P2). qRT-PCR analysis revealed that P2 strongly upregulates the expression of key genes involved in soft tissue sealing. 3) Immunohistochemical analysis of Ln-5 distribution. In the top row, the images show the gingival mucosa around the tooth and around implanted titanium abutments, either blank or coated with P1 and P2 peptides. The bottom row shows a magnification of selected areas (white square) indicated in the images above. For a complete interpretation of the figure, we refer the reader to the original publication. Reprinted from Ref. [296], with permission from Elsevier.

Kang *et* al. effectively immobilized the epidermal growth factor (EGF) on Ti surfaces via a phosphorylated Ti-binding peptide, confirming that the promotion of cell growth is correlated to the prolonged activation of cell signal transduction [293]. A similarly designed bifunctional peptide, featuring the cell attachment peptide RGDS, improved the adhesion and proliferation of osteoblasts and fibroblasts on implant-grade Ti [294]. A follow up study confirmed that the elicited cell signal transduction is needed for improved mineral deposition and osteogenesis [295]. A recent and thorough study by Liu et al. confirmed this finding using implants *in vivo* [296]. As shown in Fig. 7, the designed fusion peptide could anchor oral epithelial cells, sustain epithelial sealing *in vitro* and *in vivo* and promote the expression of key genes involved in wound healing. In a more articulated architecture, silk fibroin protein was grafted with both a Ti-binding peptide and an RGD peptide in equal ratio. The obtained silk was grafted onto a Ti surface, resulting in improved fibroblast adhesion and strongly bound endothelium [297]. In another example, an *in vivo* study showed that a fusion protein between a Ti-binding peptide and the bone morphogenic protein BMP-2 can stabilize collagen gels around implanted Ti, affecting osteoinduction in the surrounding tissue [298]. More recently, 3D printed Ti implants were coated with a Ti-binding fusion protein, promoting angiogenesis and osteointegration both *in vitro* and *in vivo* [299].

Growth of pathogenic bacteria on the surface of an implant leads to inflammation and infection. Therefore, to increase the chance of successful integration of an implanted device, bacterial growth must be minimized. For this reason, biofunctionalization mediated by SBPs has largely focused on the mitigation of bacterial biofilm formation using antimicrobial peptides (AMPs). In a first attempt, four known AMPs were fused with a Ti-binding peptide. The resulting fusion peptides could effectively coat a Ti surface, while reducing the attachment of the oral pathogen *Porphyromonas gingivalis.* [300] In a follow-up study, a rigid linker was introduced between the two peptides, improving the adsorption onto Ti surfaces and reducing the growth of *Streptococcus gordonii* and *Streptococcus sanguinis* [301]. A similar approach was explored in two related studies, where a Ti-binding peptide was fused to sequences derived from human β-defensin-3 via flexible linkers. Peptide-coated Ti substrates showed resistance to biofilm formation from *Streptococcus oralis*, *S. gordonii*, and *S. sanguinis* [302,303]. Furthermore, the addition of an RGD peptide to the design allowed for the proliferation of osteoblasts *in vitro* [303]. The efficacy of similar bifunctional designs was tested *in vitro* against *E. coli*, *Streptococcus mutans* and *Streptococcus epidermidis* [304,305]. Although the antimicrobial activity of these peptides was never tested on *in vivo* models, Wisdom et al. recently proved that such peptides can efficiently bind to Ti implantable surfaces in the presence of serum proteins and resists brushing with a commercially available electric toothbrush [306,307].

Finally, for the sake of completeness, we refer the interested reader to other successful biomedical applications of SBPs on other metal surfaces such as zinc [103,308], stainless steel [309], and iron [310].

### 3.5 Plastics

In the last decades, plastics have become ubiquitous materials for packaging, construction, clothing, and many more applications. Synthetic polymers are exceptional materials, showing durability, chemical and physical resistance, flexibility, ease of use, and low production costs. Although most plastics are petroleum-based and non-biodegradable, they are widely used for the manufacturing of laboratory and medical equipment like catheters, membranes, surgical sutures and implants [311]. Especially for these applications, surface biofunctionalization is needed to adequately modulate the interactions of plastic materials with biological fluids, cells, and tissues [73,312]. After the identification of multiple plastic-binding peptides mostly via phage display, many plastic surfaces have been successfully functionalized in recent years [73,[313], [314], [315]]. Not surprisingly, plastic-binding peptides are usually enriched in hydrophobic amino acids, while polystyrene preferably binds to peptides enriched in aromatic residues such as tryptophan (W) [76]. In the current section, we will review the application of plastic-binding peptides in vitro cell culturing and in the synthesis of smart materials.

A few simple designs have been developed to enhance the biocompatibility of plastic surfaces. For example, the epidermal growth factor was anchored to polystyrene dishes via a polystyrene-binding peptide [316]. The obtained surface was used for the adhesion and selective expansion of neural stem cells for neurosphere culture. In a similar approach, Waku et al. coated isotactic poly (methyl methacrylate) (it-PMMA) surfaces with a fusion protein featuring two plastic-binding peptides and an RGD motif for cell adhesion [317]. The protein could uniformly adsorb on it-PMMA, resulting in the effective attachment and spreading of mouse fibroblasts on the surface. In another example, polydimethylsiloxane (PDMS) surfaces were functionalized with an anchor peptide fused to the cell-adhesive GRGDS motif [318]. This one-step coating strategy allowed for the adhesion and proliferation of mouse fibroblasts and endothelial cells on PDMS. Finally, Carson et al. showed how these bifunctional peptides can be harnessed to mimic the myocardial structure *in vitro* [319]. A nanopatterned polyurethane acrylate (PUA) surface was functionalized with a polypeptide featuring a PUA-binding peptide and an RGD motif. Subsequently, human stem cell-derived cardiomyocytes were cultured on the nanogrid, resulting in mature and aligned sarcomere structures.

In more recent studies, plastic-binding peptides have been included in more sophisticated designs, providing promising results for future *in vivo* applications. For example, Garay-Sarmiento et al. exploited a plastic-binding peptide to develop a coating for wound dressings [320]. The coating consists of two hybrid constructs adsorbed on polycaprolactone (PCL) surfaces via a PCL-binding peptide: while the first construct exhibits antifouling properties thanks to a grafted polymer block, the second construct features the bactericidal enzyme endolysin. Tests conducted *in vitro* showed that the coating could effectively repel and kill *E. coli* and *Streptococcus agalactiae*, both on flat surfaces and electrospun PCL meshes. Furthermore, the coating showed antifouling properties against fibroblasts and blood plasma proteins.

**Fig. 8:**
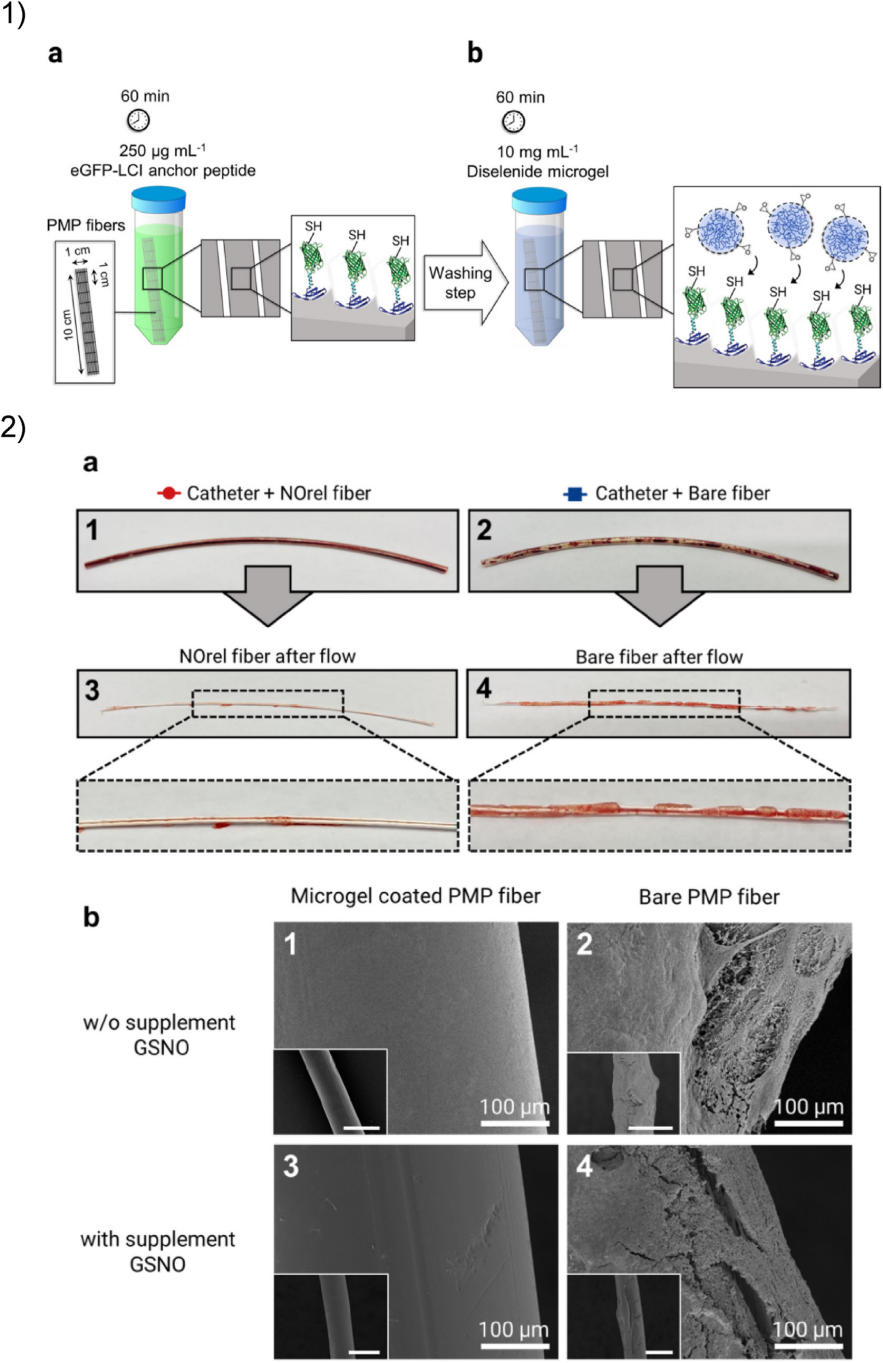
Plastic-binding peptides have been included in sophisticated designs. 1) Schematic procedure for the coating of poly (4-methylpentene) (PMP) fibres with nitric oxide-releasing microgel (NOrel). (a) The polymer-binding fusion protein is incubated with PMP fibres, (b) the microgels are immobilized to the adsorbed support via a click reaction. 2) The fibres were tested in blood flow experiments: (a) while coagulated blood can be seen in non-coated fibres, (b) SEM analysis showed that NOrel coated fibres can prevent the deposition of fibrin networks and platelet aggregation. Adapted from Ref. [322].

Another application was developed by Hosseinnejad et al. for the immobilization of an antifouling, nitric oxide-producing hydrogel for extracorporeal membrane oxygenation, as shown in Fig. 8 [321]. A plastic-binding peptide acted as anchor, allowing the facile and stable connection of the hydrogel to a hydrophobic poly (4-methylpentene) (PMP) membrane (Fig. 8.1). Thanks to the catalytic activity of the hydrogel, the membrane inhibited platelet activation and reduced clot formation when in contact with human whole blood. A follow up study from the same authors confirmed the *in vitro* functionality of the microgel coating in a PMP catheter challenged with a blood flow (Fig. 8.2) [322].

Finally, an extensive study by Hintzen et al. evaluated different plastic-binding peptides for the development of a bioadhesive coating [323]. Multifunctional fusion proteins were screened for simultaneous binding to poly (chloro-*p*-xylylene) (Parylene C) surfaces and the extracellular matrix of the retina. Subsequently, cytotoxicity tests with retinal progenitor cells indicated that a candidate protein showed good biocompatibility. An *ex vivo* proof-of-concept experiment with rabbit eyes proved the efficacy of the bioadhesive coating on Parylene C retinal stimulating arrays.

The examples featured in this section clearly show that plastic-binding peptides can be included in complex designs, with promising results for *in vivo* applications. These examples highlight how SBPs can be effectively paired to various other functionalities, warranting biocompatibility, flexibility, and control of surface chemistry.

### 3.6 Graphene

Due to their unique mechanical and electrical properties, two-dimensional carbon materials such as graphene are attractive for biological and chemical sensing applications. Graphene is a metallic and fully conducting two-dimensional crystal that has justifiably received attention in recent years. Extensive research on graphene has revealed that covalent functionalization of its surface results in defects and loss of electronic properties, making non-covalent techniques largely preferred [324]. It is therefore unsurprising that many graphene-binding peptides have been isolated and characterized for a wide variety of applications [325]. Similarly to other carbon-based materials, graphene-binding peptides are enriched in aromatic amino acids [325]. The side chains of these residues most likely interact with the graphene surface through π- π stacking. Interestingly, the position of aromatic residues within the peptide seems to increase the binding affinity for certain regions of graphene sheets [326]. In the current section, we will mostly focus on biosensing applications.

In a first sensing approach, Mannoor et al. developed a graphene field-effect transistor (GFET) biosensor for the detection of bacterial cells [327]. At first, a graphene-binding peptide was fused to the frog-derived antimicrobial peptide odorranin-HP. The fusion protein was then adsorbed onto a graphene surface and subsequently printed onto biodegradable silk fibroin. As proof-of-concept, the resulting flexible biosensor was implanted on a bovine tooth and used for the real-time remote sensing of the pathogen *Helicobacter pylori* in human saliva. Furthermore, the sensor was interfaced with an intravenous (IV) bag to simulate its usage for biohazard monitoring in hospital sanitation. Consequently, the sensor could detect *S. aureus* in the IV liquid at a reported concentration of 1 bacterium μL^−1^.

Another GFET biosensor was developed by Khatayevich et al. for the detection of biomolecules at low concentrations [328]. More recently, Kim et al. developed a GFET biosensor for the detection of the neurotransmitter neuropeptide Y (NPY) [329]. Via phage display, the authors identified an NPY-binding peptide and fused it to a graphene-binding peptide. The resulting protein was then adsorbed onto graphene and the sensor was analyzed using liquid-cell TEM. This technique allowed for the simultaneous testing of the sensor and the *in operando* observation of the sensor surface. As a result, the GFET device could detect NPY with pM sensitivity, even in presence of a competing molecule. A more recent study by the same authors reported a reduction of the sensor's sensitivity when used with artificial sweat, revealing a common hurdle of potentiometric sensing [330].

Finally, Wang et al. explored the use of graphene-binding peptides as biocompatible material synthesizers [331]. A graphene-binding peptide was connected to the cell-binding RGD peptide via a hydrophilic elastin-like polypeptide (ELP) and used to coat reduced graphene oxide (rGO). Since ELPs show interesting stimuli-responsive properties, their adsorption onto the surface was shown to modulate the hydrophobicity and aggregation of rGO. Furthermore, the resulting surface showed improved osteoblast cell attachment compared to unmodified rGO.

In conclusion, graphene-binding peptides have been successfully explored as a facile approach for modifying graphene for biomedical applications. Despite the promising proof-of-concept experiments, it appears that more research is needed for the widespread adoption of graphene in the biomedical field. We also wish to point the interested reader towards other two-dimensional materials that are being investigated, although successful biomedical applications are scarce [325,332].

## 4 Outlook

In this review, we have collected and reviewed numerous successful applications of solid-binding peptides and solid-binding proteins on biomedically relevant surfaces. We have shown that SBP-mediated bioconjugation can be easily introduced into complex designs and on nanomaterials with very different surface chemistries. The examples collected in this review show that SBPs can be adapted for use under various conditions, with good binding strength for the materials under consideration.

The use of material specific SBPs, however, does not always guarantee a direct translation from *in vitro* to *in vivo* applications. As in all physical adsorption processes, the competitive replacement from proteins contained in biological fluids (Vroman effect) can compromise the functionality of SBP coatings in physiological environments [358]. The susceptibility of protein-based coatings to protease degradation is yet another factor to evaluate, especially in long term applications [359]. As a first step, exploring various molecular architectures and testing more than one SBP can provide valuable indications on which protein design is more suitable. For example, increasing the multivalency via the oriented display of multiple SBPs might be a sufficient strategy to reduce competitive displacement. Secondly, as suggested earlier by Care et al., the isolation and selection conditions of a suitable SBP should appropriately mimic the physiological environment in which the SBP will have to operate [32]. Fortunately, combinatorial design strategies are flexible regarding the working conditions and they have been successfully introduced in alternative biopanning systems [33,[360], [361], [362]]. Moreover, the application of directed protein evolution to solid-binding peptides can compensate the shortcomings of phage display (sequence and interaction biases) and should be explored also with existing peptides.

The discovery and design of solid-binding sequences has experienced sizeable developments in the past decades. As we described in section 2.1, many techniques are available for finding functional peptide and protein sequences, leading to large data sets that we partially collected in Table 1. Nonetheless, systematic engineering of new peptides and proteins for biomedical applications is still difficult, for the following reason. As our review shows, smaller SBPs are used in biomedical applications more often than larger SBPs. Computational tools for de novo SBP design are most successful at designing large, folded solid-binding domains and proteins. Moreover, the de novo design of small sequences is severely limited by our poor understanding of surface binding and surface chemistry in aqueous environments. In these regards, the disparity between experimental quantification and molecular simulations can highly vary for different surfaces: gold and silicates, for example, are much better understood than titanium and silver. Thus, for de novo design of SBPs for medical applications, the available methods do not align with demand.

The limited characterization of surface binding is a major constraint in the design and tailoring of solid-binding sequences. In general, only a selection of known SBPs is characterized using more than one technique, while in most cases SBP binding is described only with one technique such as QCM-D or SPR. A more thorough characterization should combine different techniques. In addition, simple adsorption models are not always the correct choice for calculating affinity constants. Since SBPs often do not bind independently and can form surface assemblies, a quantitative and qualitative estimate of surface coverage is necessary to better elucidate the binding process [79,282,363].

Unfortunately, there is very little work performing detailed physical characterization of larger sets of SBPs, which would allow for a fair comparison between the performance of different SBP sequences. Instead, for each material, a few well characterized and validated sequences are used in many studies. This approach can obviously yield positive results, but also introduces a certain bias. A recent report on gold-binding peptides suggests that the binding affinity of many sequences is in fact quite comparable [23].

In conclusion, solid-binding peptides, domains, and proteins are useful surface functionalization tools for the design of novel biomaterials and in the construction of novel biomedical devices. Despite the theoretical and technological challenges, the promising results highlighted in our review clearly show that SBPs can provide a convenient alternative to covalent functionalization strategies. On the other hand, many of the applications do still seem to be in the research phase and, to the authors’ knowledge, not many are already being used in commercial applications. This observation suggests that significant technical hurdles still exist, as implicitly hinted in the current literature. For the field to achieve maturity and real translations to commercial applications, it is crucial that such hurdles are made more explicit in the literature and are being considered as key research issues in the field.

As we have previously outlined, one such hurdle may be that quantitative understanding and design of SBPs as well as standardization of characterization data are not well developed. This makes it hard to compare and systematically improve different SBPs. Another hurdle is that in many cases the affinity of single SBPs may not be enough for stable attachment, therefore strategies for the multivalent use of SBPs should be given more attention.

Nevertheless, the ease of use of SBPs is a key advantage for their application to new or underexplored materials that might benefit from the flexibility offered by non-covalent functionalization. Although we could not include all biomedically relevant surfaces in our review, application-driven designs could and should explore still more materials such as biodegradable plastics, absorbable polymers, 2D nanostructures, and gold and silver nanoclusters.

## Declaration of competing interest

The authors declare that they have no known competing financial interests or personal relationships that could have appeared to influence the work reported in this paper.

## Data Availability

No data was used for the research described in the article.
